# Immune Checkpoint Inhibitors in Prostate Cancer

**DOI:** 10.3390/cancers13092187

**Published:** 2021-05-02

**Authors:** Shobi Venkatachalam, Taylor R. McFarland, Neeraj Agarwal, Umang Swami

**Affiliations:** 1 Department of Internal Medicine, Nazareth Hospital, Philadelphia, PA 19152, USA; shobi.venkatachalam@mercyhealth.org; 2 Department of Internal Medicine, Huntsman Cancer Institute, University of Utah, Salt Lake City, UT 84112, USA; Taylor.McFarland@hci.utah.edu (T.R.M.); Neeraj.Agarwal@hci.utah.edu (N.A.)

**Keywords:** immune checkpoint inhibitors, prostate cancer

## Abstract

**Simple Summary:**

Metastatic prostate cancer is an incurable disease with limited treatment options. Immunotherapy has demonstrated significant success in multiple cancer types but efforts to harness its benefit in prostate cancer have so far largely been unsuccessful. In this review, we analyze the preclinical rationale for the use of immunotherapy and underlying barriers preventing responses to it. We summarize clinical studies evaluating checkpoint inhibitors in prostate cancer. In the end, we review ongoing trials exploring combination immune checkpoint inhibitors in combination with other agents with the intent to modulate the immune system to improve treatment outcomes.

**Abstract:**

Metastatic prostate cancer is a lethal disease with limited treatment options. Immune checkpoint inhibitors have dramatically changed the treatment landscape of multiple cancer types but have met with limited success in prostate cancer. In this review, we discuss the preclinical studies providing the rationale for the use of immunotherapy in prostate cancer and underlying biological barriers inhibiting their activity. We discuss the predictors of response to immunotherapy in prostate cancer. We summarize studies evaluating immune checkpoint inhibitors either as a single agent or in combination with other checkpoint inhibitors or with other agents such as inhibitors of androgen axis, poly ADP-ribose polymerase (PARP), radium-223, radiotherapy, cryotherapy, tumor vaccines, chemotherapy, tyrosine kinase inhibitors, and granulocyte-macrophage colony-stimulating factor. We thereafter review future directions including the combination of immune checkpoint blockade with inhibitors of adenosine axis, bispecific T cell engagers, PSMA directed therapies, adoptive T-cell therapy, and multiple other miscellaneous agents.

## 1. Introduction

Globally, in 2020, prostate cancer was the second most common cancer and the fifth leading cause of cancer-related deaths among men [1]. Once metastatic, it is incurable. Apart from androgen deprivation therapy (ADT) which is the backbone of the management of metastatic prostate cancer, treatment options mainly consist of either novel hormonal therapies (NHT; abiraterone, enzalutamide, apalutamide) or taxane-based chemotherapy (docetaxel and cabazitaxel). Other treatment options are restricted to a certain subset of metastatic prostate cancer patients that are castrate resistant. For example, sipuleucel-T is recommended for asymptomatic or minimally symptomatic patients with no liver metastasis, radium-223 is recommended only for patients with symptomatic bone metastasis and no visceral metastasis while olaparib and rucaparib are recommended only for patients with selected 14 sensitizing homologous recombination repair (HRR) and BRCA 1/2 mutations respectively [2,3]. Given the limited treatment options for the majority of patients and the attractive success of immune checkpoint inhibitors (ICI) in other advanced cancers such as melanoma and lung cancer; an increasing focus on treating prostate cancer with ICI is being made [4,5].

## 2. Biological Rationale and Barriers to Immune Checkpoint Blockade in Prostate Cancer

PD-1 is expressed on activated T cells, B cells, and natural killer (NK) cells and it has two ligands: programmed death-ligand 1 (PD-L1) and programmed death-ligand 2 (PD-L2). The binding of PD-L1 to PD-1 inhibits pathways involved in T cell activation and converts naive T cells to regulatory T cells, thus keeping the immune system from overzealously destroying the normal cells during antigen-specific responses [6,7,8]. Tumor cells by expressing PD-L1 evade the T cell antitumor response through anergy, or apoptosis of the effector T cells. CD28 and CTLA-4 are present on T cells like PD-1 and bind to ligands CD80 and CD86. Interaction of CD28 with these ligands activates T cells but when CTLA-4 binds to these ligands it inhibits T-cell stimulation [9].

Multiple preclinical studies have investigated PD-1/L1 expression in prostate cancer specimens to evaluate the rationale of treatment with checkpoint inhibitors in these patients. The results are summarized in Table 1. These studies lacked a uniform criterion for determining PD-L1 positivity which partly explains the differences in results from these studies. For example, in a study utilizing immunohistochemistry (IHC) tumor scoring of 402 prostatectomy specimens, 92% (371/402) of cases were positive for PD-L1 staining in tumor epithelial cells and 59% (236/402) cases had a high PD-L1 intensity score. While a high density of PD-1 + lymphocytes was significantly associated with shorter clinical failure-free survival, no significant association between PD-L1 expression and prostate cancer outcomes was observed in this study [10]. In another study involving primary prostate cancer specimens from 2 different cohorts, 50% to 60% of cases expressed moderate to high levels of PD-L1 on IHC staining on an average. There was a positive correlation between PD-L1 expression, proliferation (Ki-67), and Gleason score. Also, PD-L1 positivity was prognostic for biochemical recurrence on multivariate cox analysis in this study (*p* = 0.007; Hazard ratio-1.46) [11]. In contrast, in another study, only 3 of the 20 primary prostate cancer samples (15%) were PD-L1 positive where PD-L1 “positivity” was defined as 5% membrane staining [12]. Furthermore, about 19% of patients in another series of 16 patients with castrate-resistant prostate cancer (CRPC) showed high PD-1/PD-L1 immunoscores [13]. In yet another series involving prostatectomy/biopsy tissues from 25 men with high-grade prostate cancer only about 8% scored high for PD-1/PD-L1 expression [14].

There are several nuances to using immune checkpoint blockade therapy in prostate cancer. Prostate cancer is immunologically cold with a low tumor mutation burden (TMB) which is about 7–15 times lower than melanoma or lung cancer [15]. This translates to a lower number of immune cell attractions including T cells into the tumor tissue. Also, the T cell infiltration into the tumor tissue is poor secondary to hypoxic zones within the prostate cancer. These hypoxic zones render the tumor microenvironment non-congenial for the T cells by a variety of mechanisms including acidic pH, the depletion of essential nutrients, abnormal angiogenesis, increased expression of adenosine, T-cell inhibitory PD-L1, and immunosuppressive transforming growth factor-Beta (TGF-B) [16,17]. Low CD8+ T cell infiltration in turn translates to poor response to immune checkpoint blockade [18]. Also, hypoxic zones promote the phenotypic conversion of immature myeloid cells to myeloid-derived suppressor cells (MDSCs) and tumor-associated macrophages making the tumor environment even more immunosuppressed [16].

**Figure 1:**
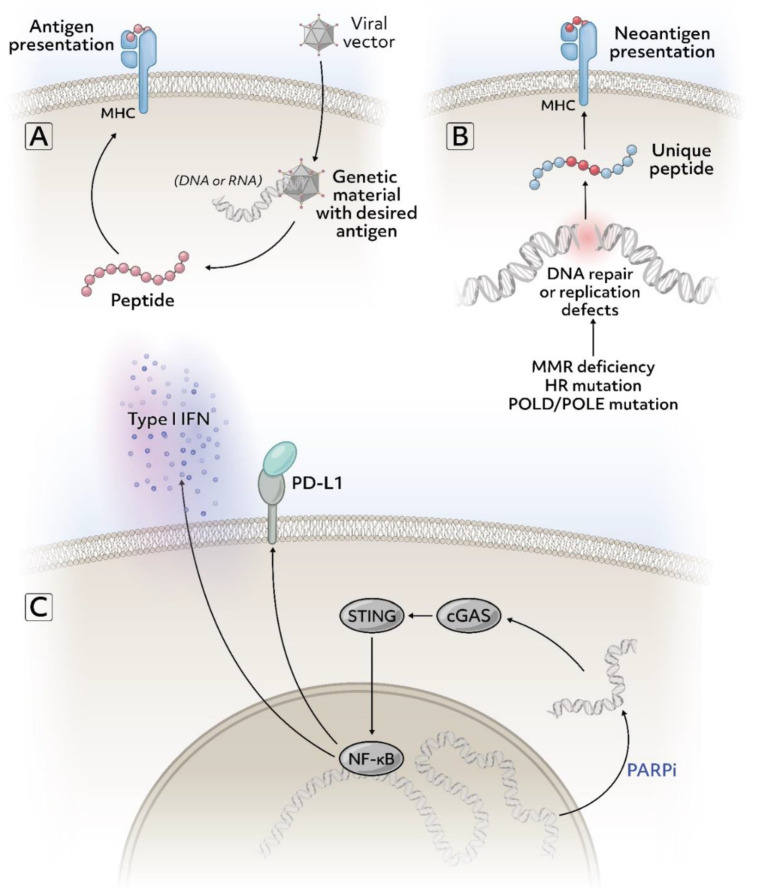
Select mechanisms to target immune pathways in prostate cancer (**A**) Viral vector from a vaccine containing a sequence for antigen presentation such as prostate-specific antigen or other targets that may be enriched in prostate cancer. (**B**) Many mutations commonly found in prostate cancer cause DNA repair deficiency or replication defects and lead to more mutations. If these mutations result in changes to the amino acid sequence of a protein, they can serve as potential tumor-specific neoantigens. (**C**) Treatment with poly-ADP (ribose) polymerase inhibitors (PARPis) can cause DNA to leak into the cytoplasm and trigger the cGAS-STING pathway which can induce an immunostimulatory response. Figure created via Adobe Inc. (2021). Adobe Illustrator version 25.2.3. Retrieved from https://adobe.com/products/illustrator (accessed on 11 April 2021).

**Figure 2:**
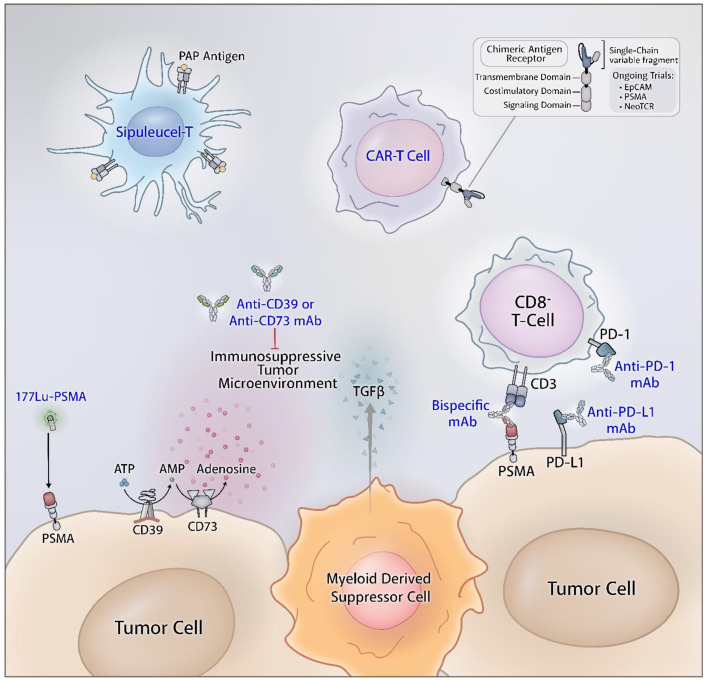
The immune microenvironment of prostate cancers. Myeloid-derived suppressor cells, increased adenosine concentrations, and immune checkpoints promote an immunologically cold phenotype. Monoclonal antibodies that target these proteins can help reduce immunosuppression. Cell-based such as sipuleucel-T and chimeric antigen receptor (CAR) T cell therapies can be engineered to target specific aspects of the tumor. Figure created via Adobe Inc. (2021). Adobe Illustrator version 25.2.3. Retrieved from https://adobe.com/products/illustrator (accessed on 11 April 2021).

At the cellular level, the T cell population in prostate cancer largely consists of CD4+ FOXP3+ CD25+ T cells and CD8+ FOXP3+ CD25+ T cells. FOXP3+ T cells are regulatory T cell subsets that are immunosuppressive by inhibiting naive T cell proliferation and by producing inhibitory cytokines [19,20]. At the molecular level, the expression of major histocompatibility complex (MHC) class I, a molecule presenting antigenic protein fragments to cytotoxic T cells are lost or diminished in prostate cancer [21,22]. Also, PTEN is frequently lost which has been found to adversely affect the tumor microenvironment and subsequently the response to immunotherapy [23]. At the cytokine level, chronic activation of the interferon-1 (IFN-1) pathway associated with PTEN loss has been demonstrated in prostate cancer studies which have immunosuppressive effects in contrast to the usual IFN-1 associated immunostimulatory and anti-tumor effects [24]. Table 2 presents selected clinical trials evaluating immunotherapy in prostate cancer and Figure 1 and Figure 2 present underlying mechanisms of action of these agents.

## 3. Predictors of Response to Immune Checkpoint Blockade

Though PD-L1 expression on tumor cells and stromal cells within the tumor may predict favorable responses to PD-1/PD-L1 blockade therapy, this is not always true. There exists considerable intratumoral heterogeneity with regards to PD-L1 expression along with inter-assay variability, limiting PD-L1 expression as the sole predictor of response to PD-1/PD-L1 blockade [49]. PD-L1 expression in the tumor is not static as it may increase with tumor progression [50]. Also, PD-L1 expression can be modulated by radiation and chemotherapy [51,52,53,54]. Moreover, concomitant genomic alterations such as homologous recombination deficiency (BRCA2, ATM, CDK12 mutations), microsatellite instability-high (MSI-H) or mismatch repair-deficiency (dMMR), and POLE/POLD1 mutations can increase the responsiveness to ICI by increasing the tumor mutation burden (TMB) and expression of neoantigens [55].

Although prostate cancer is generally considered to be an immunologically cold cancer with only between 50–100 nonsynonymous DNA alterations per cancer exome (i.e., 1–2 mutations per Mb), germline or somatic mutations in DNA repair genes especially homologous recombination (HR) repair genes (BRCA2, ATM, etc.) have been uncovered in a significant percentage of metastatic castration-resistance prostate cancer (mCRPC) patients. Defects in these DNA repair genes can increase TMB and neoantigen load potentially predicting response to immunotherapy [56,57]. In a study involving a cohort of 4129 prostate cancer patients 1.8% (74/4129) of patients had POLE/POLD1 mutations. The TMB of patients with these mutations was significantly high compared with patients without these mutations suggesting that these patients might benefit from ICI. Based on this rationale, a phase 2 study of toripalimab (a PD-1 antibody) in patients with advanced solid organ tumors including prostate cancer and POLE/POLD1 positive status has been initiated [58].

In an analysis of 360 mCRPC patients, the loss of cyclin-dependent kinase (CDK12) that controls DNA damage response) was seen to be associated with focal tandem duplications, increased gene fusion, neoantigen burden, and T cell infiltrations, suggesting that this subset of prostate cancer patients might benefit from immune checkpoint inhibition [59,60]. In another study of 1033 patients with adequate tumor quality, only 32 (3.1%) had microsatellite instability or mismatch repair deficiency, and 21.9% (7/32) of these had Lynch syndrome-associated germline mutations. Also, of the six patients who had tumor analysis more than once, two (33%) demonstrated an acquired MSI-H phenotype later in their disease course. Among the eleven patients with microsatellite unstable or mismatch repair deficient CRPC who received anti-PD-1/PD-L1 therapy, 54.5% (6/11) had a PSA response, and 66% (4/6) of these patients also had a radiographic response [61].

PD-L1/PD-L2 positivity in dendritic cells (DCs) of patients who had progressed on enzalutamide is increased compared to patients who were enzalutamide naive or who had responded to enzalutamide [62]. Androgen ablation also upregulates adaptive immunity in prostate cancer by increasing naive T cell expansion [63]. In a phase II trial of 28 men with mCRPC treated with pembrolizumab and enzalutamide after progressing on enzalutamide, a PSA response was obtained in about 18% of patients, and an objective response in 25% (3/12) of patients who had measurable disease. None of the three responders had detectable PD-L1 expression [64].

## 4. Studies Evaluating Single Agent CTLA-4 Inhibitors in mCRPC

In phase III CA184-095 trial, high dose ipilimumab (10 mg/kg) monotherapy did not show an improvement in median OS compared to the placebo (28.7 months versus 29.7 months; HR = 1.11, 95% CI 26.1–34.2 months, *p* = 0.3667) in chemotherapy naïve minimally symptomatic mCRPC patients. But higher median progression-free survival (5.6 months vs. 3.8 months; HR = 0.67, 95.87% CI 0.55–0.81), PSA response rates (23% vs. 8%), and longer time to systemic nonhormonal cytotoxic therapy were observed compared to placebo, indicating antitumor activity. More treatment-related grade 3 to 4 adverse events (TRAEs) were observed compared to the 3 mg/kg dose used in melanoma (40% vs. 23%) and there were 9 treatment-related deaths (comparable to prior studies) [46].

## 5. Studies Evaluating Single Agent PD-1/L1 Inhibitors in mCRPC

KEYNOTE-028, a phase Ib study has reported an objective response rate (ORR) of 17.4% (95% CI: 5.0–38.8%) with pembrolizumab in a cohort of 23 heavily pretreated mCRPC patients with measurable disease and ≥1% PD-L1 expression in tumor or stromal cells. The response was a partial response (PR) in 4 patients and 3/4 experienced parallel biochemical response (defined as >50% PSA decline from baseline) [36]. Following the favorable side effect profile (no deaths or treatment discontinuations because of TRAEs) in the KEYNOTE-28 trial, pembrolizumab has been subsequently studied as a monotherapy or in various combinations.

The KEYNOTE-199 trial evaluated the activity of pembrolizumab as monotherapy in three mCRPC cohorts. Cohort 1 enrolled patients with PD-L1 positive tumor and measurable disease, cohort 2 enrolled PD-L1 negative tumors and measurable disease, while cohort 3 enrolled non-measurable, bone metastatic disease regardless of the PD-L1 status. Median OS was 9.5 months (6.4 to 11.9 months; 5% CI), 7.9 months (5.9 to 10.2 months; 95% CI), 14.1 months (10.8 to 17.6 months; 95% CI) and confirmed PSA response was 6% of 124 patients, 8% of 60 patients, and 2% of 59 patients in cohorts 1, 2, and 3, respectively. Observed ORR was modest (about 5%), with a median duration of 16.8 months and 55% (5/9) had ongoing responses at data cutoff. Other interesting observations in this trial were similarity of outcomes regardless of PD-L1 status (combined positive score ≥1 was used to define positivity) and no clear relationship between responses to pembrolizumab and DNA damage repair (DDR) gene mutation status as determined by whole-exome sequencing [40].

Atezolizumab, a PD-L1 antibody as monotherapy has shown favorable safety and clinical activity with no grade 4-TRAEs and a 55.6% 12-month OS (95% CI: 27.4, 83.7). The median OS was still not reached during data cut off (range, 2.3–23.0 months) in these 15 heavily pretreated mCRPC patients [27].

## 6. PD-L1 Blockade in Combination with Androgen Inhibitors

The IMbassador 250 trial randomized 759 patients with mCRPC to atezolizumab with enzalutamide or enzalutamide alone after they had progressed on an androgen synthesis inhibitor therapy. The combination arm failed to demonstrate any significant improvement in the overall survival rate (12 months OS 64.7% vs. 60.6%), ORR, PSA response rate, or radiographic progression-free survival (rPFS) compared to the control arm [28]. This was despite preclinical studies showing signals for improved responses from immune checkpoint blockade via enzalutamide-induced enhanced IFNγ pathways [65].

In another study, enzalutamide in combination with pembrolizumab in 102 patients with mCRPC (KEYNOTE 365, COHORT C) showed a PSA response rate of 22% and ORR of 12% (based on RECIST 1.1, in those with measurable disease). All responses lasted ≥12 months and the median duration of response (DOR) was not reached. Ninety percent of the study participants had TRAEs and there was one treatment-related death [39].

Finally, the KEYNOTE 199 study examined the safety and antitumor efficacy of enzalutamide plus pembrolizumab combination after enzalutamide progression in patients with RECIST-measurable disease (cohort 4, *n* = 81) or bone predominant disease (cohort 5, *n* = 54). The ORR was 12% in cohort 4 with 2 complete responses (CR) and 8 PR’s. The 12-month overall survival rate in the cohort 4 and 5 were 70% vs. 75% respectively and the median OS was not reached vs. 19 months, respectively. Liver metastasis and a shorter period of enzalutamide treatment (<6 months) prior to progression were associated with shorter median OS [41].

## 7. Immune Checkpoint Blockade with PARP Inhibitors

Poly ADP-ribose polymerase (PARP) inhibition can potentiate responses to PD-1/PD-L1 inhibition via a number of mechanisms including increased TMB secondary to unrepaired DNA damage (especially in patients with DDR gene mutations), enhanced PD-L1 expression, and immune cell infiltration into the tumor microenvironment. (Figure 1) [66,67]. In the durvalumab plus olaparib trial involving 17 mCRPC patients after progression on androgen receptor blockade therapy, median rPFS for all patients was 16.1 months (95% CI: 4.5–16.1 months), 53% (9/17) patients had a PSA decline of ≥50% and 4/9 patients had radiographic response per RECIST v.1.1. Patients with mutations in DDR genes responded better with an 83.3% probability of 12-month progression-free survival (PFS) compared to 36.4% in those without mutations [25]. Similarly, olaparib with pembrolizumab in molecularly unselected mCRPC patients (KEYNOTE-365, cohort A) showed an OS of 14 months (95% CI: 8–19), PSA response rate of 9%, and ORR of 8% with 2 partial responses. Both responses lasted ≥12 months and the median response duration was not reached at the time of data reporting [37].

## 8. Immune Checkpoint Blockade with Radiotherapeutic Agents, Radiotherapy, or Cryotherapy

Radium-223 dichloride (radium-223) is an alpha-particle emitting radiotherapeutic agent that accumulates preferentially in areas of high bone turnover such as bone metastasis and has shown to improve OS in mCRPC patients with bone metastasis [68]. A phase Ib study evaluated the safety and tolerability of atezolizumab plus radium-223 in 44 patients. Though no new safety concerns were encountered with this combination beyond that already known with atezolizumab and radium-223, the combination failed to show a clinical benefit ORR 6.8% (95% CI: 1.43, 18.66). The median radiological PFS was 3.0 months (95% CI: 2.8, 4.6) and median OS was 16.3 months (95% CI: 10.9, 22.3) [48].

Radiotherapy through systemic antitumor effects can cause tumor regression at sites distant from the primary site (abscopal effect). In murine models, tumor irradiation when combined with an anti-CTLA-4 antibody has demonstrated synergistic systemic antitumor effects and metastasis inhibition [69,70]. Based on this, an escalating dosage of ipilimumab with or without radiotherapy was evaluated in patients with mCRPC. Among 28 evaluable patients in this study who received 10 mg/kg ipilimumab with or without radiotherapy, one had a complete response, and 6 had stable disease. Sixteen percent of patients (8/50) had ≥50% PSA decline [45]. CA184-043, a phase III randomized trial compared ipilimumab against placebo following radiotherapy in 799 mCRPC patients (randomized 1:1) who had progressed on docetaxel therapy. The median OS was similar (11.2 months with ipilimumab vs. 10.0 months with placebo; HR: 0.85, 0.72–1.00; *p* = 0.053) in intention-to-treat patients [47]. However, a difference in OS rates was observed on longer follow-ups. The OS rates in the ipilimumab arm compared to the placebo arm at 2 years were 25.2% vs. 16.6% and up to 7.9% vs. 2.7% at 5 years respectively [71]. In addition, median OS was 22.7 months with ipilimumab compared to 15.8 months with placebo in patients with favorable prognostic findings like alkaline phosphatase levels less than 1.5 times the upper normal limits, hemoglobin of ≥10 g/L, and absence of visceral metastases. Major grade 3 irAEs were diarrhea, colitis, and transaminitis, and about four deaths were attributed to ipilimumab therapy [47].

Cryotherapy can also potentially induce an abscopal effect in combination with immunotherapy [72]. In a pilot study of pembrolizumab (6 doses) in combination with cryotherapy to prostate and eight months ADT, median PFS was 14 months and PSA responses were 92% (11/12) in newly diagnosed oligo-metastatic prostate cancer patients. No grade ≥ 3 AEs were reported in these 12 patients [35].

## 9. Immune Checkpoint Blockade with Tumor Vaccines

Considering that clinically meaningful responses may not be seen with ICI monotherapy alone in metastatic prostate cancer, ICI has been explored in combination with other agents such as tumor vaccines. Atezolizumab in combination with sipuleucel-T (a vaccine based on autologous antigen-presenting cells targeting prostatic acid phosphatase) was studied in 37 patients with asymptomatic or minimally symptomatic progressive mCRPC. PFS was 8.2 months in arm 1 (atezolizumab followed by sipuleucel-T) as compared to 5.8 months in Arm 2 (sipuleucel-T followed by atezolizumab) (*p* = 0.054). OR by RECIST at 6 months was SD in 41% (10/24) and PR in 8% (2/24) of patients. No grade 3 or 4 irAEs occurred but twelve grade 3 TRAEs and two grade 4 TRAEs were noted [30].

ChAdOx1-MVA 5T4, a virally vectored vaccine designed to produce the tumor antigen 5T4, after it demonstrated safety and T cell responses in the VANCE trial [73], was studied in combination with nivolumab in the ADVANCE trial. Preliminary results from this trial showed a PSA response (>50% reduction in PSA level) in 22% of the patients at any time point compared to their baseline and the therapy was well tolerated [34]. Similarly, PSA-Tricom (a vector-based vaccine targeting PSA) was studied in combination with Ipilimumab and GM-CSF. This was based on the rationale that cancer vaccines induced antigen-specific T-cells to upregulate CTLA4, a negative regulatory molecule, and that CTLA4 blockade can prevent this and enhance T-cell-mediated immune responses to the vaccine. In this study, 58% (14/24) of the chemotherapy-naïve and 16% (1/6) of the patients with prior chemotherapy had a PSA decline from their baseline. Overall, 6 of 14 chemotherapy-naïve patients had >50% PSA decline and median OS was 34.4 months for all patients. Among 6 of 9 patients who could be assessed for PSA-specific T-cell responses, only a minority had significant PSA declines. And, though most common adverse effects were grade 1 or 2, about 27% (8/30) of patients had grade 3–4 side effects. Also, responses to tumor-associated antigens not incorporated in the vaccine were seen [44].

## 10. Immune Checkpoint Blockade with Chemotherapy

Chemotherapy by killing tumor cells increases tumor neoantigens, disrupts immune-suppressive pathways, and enhances effector T cell responses [74,75,76]. This suggests possible improved responses with a combination of chemotherapy and ICI therapy. In 41 chemotherapy-naive mCRPC patients treated with nivolumab plus docetaxel (CheckMate 9KD, cohort B) combination, the ORR was 36.8% (95% CI: 16.3–61.6) with one CR and six PRs and the confirmed PSA response rate was 46.5% (95% CI: 30.7–62.6) [33]. Similarly, in the KEYNOTE-365 trial (cohort B) chemotherapy plus ICI blockade (pembrolizumab + docetaxel and prednisone), ORR based on RECIST 1.1 was 18% (7/39) with 7 PRs, 5/7 (71%) of responses lasted ≥6 months with median DOR of 6.7 months range (3.4–9.0+) and the PSA response rate was 28% Also, radiological PFS was 8.3 months (95% CI: 7.6–10.1) and OS was 20.4 months (16.9-not reached) [38].

## 11. CTLA-4 and PD-1/PD-L1 Combination Therapy

Combined CTLA-4 and PD-1 blockade has been associated with more antitumor responses, one possible rationale being ipilimumab therapy increases tumor-infiltrating T cells and upregulates PD-1/PD-L1 inhibitory pathway in a compensatory fashion indicating that combination therapy may be more efficient [77,78]. Also, patients with AR-V7 isoform of the androgen receptors are less responsive to second-generation hormonal agents (abiraterone and enzalutamide) and taxanes but may have more frequent DNA-repair gene mutations and a higher mutation load making them more susceptible to treatment with ICI blockade [79,80,81]. Based on these observations, 15 patients with mCRPC expressing AR-V7 were treated with nivolumab plus ipilimumab combination (STARVE-PC). Encouraging results were seen in the subset with DDR gene mutations, but not in the overall study. The PSA response rate, ORR, and OS in the 2 subsets were 33% vs. 0% (*p* = 0.14), 40% vs. 0% (*p* = 0.46) and 9.04 vs. 7.23 months (HR 0.41; *p* < 0.01) respectively. Also, there was more PD-L1 positivity among DDR mutation-positive tumors compared with DDR negative tumors [31]. In another study with 2 cohorts of 90 pre-chemotherapy (n = 45) and post-chemotherapy (*n* = 45) mCRPC patients treated with combined ipilimumab and nivolumab (CheckMate 650), ORR, PSA response, and median OS were 25% vs. 10%, 17.6% vs. 10% and 19.0 vs. 15.2-months, respectively. Four treatment-related deaths were observed and patients with higher TMB, homologous recombination deficiency (HRD)-positive status, DDR-positive status, and PD-L1 ≥ 1% had better response rates [32].

Based on the rationale that PD-L1 is overexpressed by the dendritic cells of mCRPC patients who progress on androgen receptor antagonist therapy [62], 52 patients who had progressed on prior abiraterone and/or enzalutamide were randomized to either durvalumab alone or durvalumab (PD-L1 inhibitor) plus tremelimumab (CTLA-4 inhibitor). Patients in the combination arm had more ORR compared to the monotherapy arm [16% (95% CI: 6–32%) vs. 0% (95% CI: 0–25%)], indicating that durvalumab alone may not show enough clinical activity but the combination with PD-L1 and CTLA-4 blockade may result in better treatment efficacy. The most common TRAEs were grade 2 or less and the most common grade 3/4 TRAEs were diarrhea and elevated transaminitis. There was no grade 5 TRAEs [26]

## 12. Tyrosine Kinase Inhibitors with Immune Checkpoint Blockade

The COSMIC-021 trial evaluated the combination of cabozantinib with atezolizumab in solid organ cancers after cabozantinib showed encouraging responses in combination with ICI therapy in hepatocellular cancer and renal cell cancer [82,83]. Among 44 mCRPC patients in cohort-6 of this trial, ORR per RECIST 1.1 was 32% and 48% of patients (21/44) had SD resulting in an 80% disease control rate. The side effects were tolerable with minimal grade 3/4 events. The responses were durable and their median duration was 8.3 months [29].

## 13. Other Combinations with Immunecheck Point Blockade

Increasing doses of ipilimumab and fixed-dose GM-CSF combination were evaluated in 24 mCRPC patients based on the rationale that GM-CSF increases circulating antigen-presenting cells (APCs) including the numbers of Fc receptor-bearing cells, thereby enhancing the efficacy of another antibody drug-like ipilimumab [84]. This combination demonstrated a 12.5% (3/24) PSA response (>50% decline in PSA level), one (1/3) had PR by RECIST of the liver metastasis and another had a durable PSA response that was ongoing at almost 2 years since therapy. An increase in T cell activation markers (CD25 and CD69, especially at higher dose levels of ipilimumab), IgG antibodies to NY-ESO-1 (a tumor antigen), and interferon-γ (IFNγ) producing T cells in response to NY-ESO-1157–165 following Ipilimumab and fixed-dose GM-CSF combination treatment were seen in this study [42].

## 14. Future Directions

### 14.1. Combination Immune Checkpoint and Adenosine Axis Blockade

Adenosine has immunosuppressive and tumor-promoting effects on the tumor microenvironment. Currently, there has been a lot of enthusiasm on the blockade of the adenosine pathway as an immunomodulatory therapy either by blocking the adenosine generating enzymes (CD38, CD39, and CD73) or via antagonism of adenosine receptors (A2AR and A2BR) based on preclinical data for efficacy [85,86]. The combination of immune checkpoint and adenosine axis blockade is also being studied (ClinicalTrials.gov Identifiers: NCT04381832, NCT03629756, NCT03454451, NCT04306900, NCT03549000, NCT02655822, and NCT03367819) based on observations that upregulation of CD38 is a mechanism for acquired resistance against PD-1/PD-L1 blockade [87,88,89].

### 14.2. Bispecific T Cell Engager and Immune Check Point Blockade

Bispecific T cell engagers (BITE) by simultaneously binding to tumor antigens and T cells, bridge tumor cells with cytotoxic T cells; this, in turn, results in tumor-directed T cell activation and tumor cell lysis [90]. Recent evidence suggests encouraging activity and safety with prostate-specific membrane antigen (PSMA) directed BITE therapy as well as augmentation of response to BITE therapy with the combination of immune checkpoint blockade [91,92,93]. Based on this, AMG 160 (a bispecific T cell engager that binds to the prostate-specific membrane antigen on tumor cells and CD3 on T cells) has been studied in combination with AMG 404 (a PD-1 monoclonal antibody; ClinicalTrials.gov Identifier: NCT04631601) in one trial and in combination with pembrolizumab (ClinicalTrials.gov Identifier: NCT03792841). In the ClinicalTrials.gov Identifier: NCT03792841 trial, interim results of the monotherapy arm (AMG 160 only) involving 43 patients with PSMA positive mCRPC showed that, 27.6% of patients had a confirmed PSA response, 13.3% had a confirmed PR and 53.3% had SD with BITE therapy targeting PSMA. No grade 5 events or treatment discontinuation from TRAE were reported [94]. Also, XmAb^®^22841 (a bispecific antibody that simultaneously targets immune checkpoint receptors CTLA-4 and LAG-3 to promote tumor-selective T-cell activation) has been evaluated in the DUET-4 trial in combination with pembrolizumab (ClinicalTrials.gov Identifier: NCT03849469).

### 14.3. Lu-PSMA-617 and Immune Checkpoint Blockade

PSMA is membrane glycoprotein, which is specific to prostate cells and its expression is drastically increased in prostate cancer. Lu-PSMA-617 is a radiopharmaceutical where lutetium-177 is conjugated to the ligand PSMA-617. This combination enables direct delivery of radiation to prostate cancer cells [95,96,97]. In a phase 2 trial of 30 men with mCRPC treated with PSMA-targeted radioligand therapy, 57% (17/30) achieved a PSA response (PSA decline ≥50%) and eighty-two percent (14/17) of patients had an objective response [98]. Also, evidence supports enhanced efficacy of PSMA directed radionuclide therapy with immune checkpoint blockade [99], and based on such data Lu-PSMA-617 is being studied with pembrolizumab in the PRINCE trial (ClinicalTrials.gov Identifier: NCT03658447).

### 14.4. Adoptive T Cell Therapy and Immune Checkpoint Blockade

Adoptive T cells are tumor-specific T cells that are isolated from the patient, expanded ex vivo, and reinfused back into the patients [100]. NeoTCR-P1 is a form of adoptive T cell therapy where apheresis-derived T cells are engineered to express an autologous T cell receptor (TCR) of the native sequence. These T cells can then target a neoepitope that is unique to the patient’s tumor cells and presented in association with human leukocyte antigen (HLA) receptors. NeoTCR-P1 has been studied in combination with nivolumab (ClinicalTrials.gov Identifier: NCT03970382) based on signals that this combination may have meaningful activity [101,102].

### 14.5. Miscellaneous Agents

Other interesting combinations being studied alongside immune checkpoint blockade include fecal microbiota transplant (ClinicalTrials.gov Identifier: NCT04116775), vascular endothelial growth factor (VEGF) receptor inhibitors (ClinicalTrials.gov Identifier: NCT02484404), Valemetostat (EZH1/2 Dual Inhibitor; ClinicalTrials.gov Identifier: NCT04388852), DF6002 (a monovalent IL-12 immunoglobulin Fc fusion protein; ClinicalTrials.gov Identifier: NCT04423029), TPST-1120 (a peroxisome proliferator-activated receptor alpha antagonist; NCT03829436), Poly ICLC (a synthetic double-stranded RNA complex that is a toll-like receptor-3 and MDA-5 ligand; ClinicalTrials.gov Identifier: NCT02643303), ALT-803 (a recombinant IL15 Complex; ClinicalTrials.gov Identifier: NCT03493945), M7824 (a fusion protein with two extracellular domains of TGF-βRII and a PD-L1 monoclonal antibody; ClinicalTrials.gov Identifier: NCT03493945), GB1275 (CD11b modulator; NCT04060342), Talabostat Mesylate (a small molecule inhibitor of dipeptidyl peptidases; NCT03910660) and Vibostolimab (a monoclonal antibody, that binds to the T-cell immunoreceptor and blocks its interaction with its ligands; NCT02861573) (Table 3).

## 15. Conclusions

Though immune checkpoint blockade shows considerable preclinical activity, real-world experiences are not convincing especially with ICI monotherapies. Overall, the prospective role of immune checkpoint blockade therapy in prostate cancer awaits the results of the phase 1/phase 2 trials exploring ICI therapy in combination with a variety of immunomodulating agents (Table 3) as well as the discovery of predictive biomarkers.

## Figures and Tables

**Table 1:** Studies examining PD-1/PD-L1 expression in prostate cancer.

Specimen Type	Number of Patients	Cut Off for Positivity	Antibody/Clone Used to Detect PD-L1	PD-L1 Expression
Primary prostate cancer [10]	402	No staining = 0, weak staining = 1, moderate staining = 2, and strong staining = 3. PD-L1+ stromal cells and PD-1+ lymphocytes were scored as number of positive stained cells per 0.6 mm diameter core as follows: 0 = 0–3, 1 = 4–10, 2 = 11–15, and 3 ≥ 15	Rabbit monoclonal PD-L1 antibody (Cat#13684, clone: E1L3N, Cell signaling technology, Danvers, MA, USA)	92% (371/402) of patients were positive for PD-L1 staining in tumor epithelial (TE) cells and 59% (236/402) had high PD-L1 intensity score. Also, 66% (267/402) of patients had PD-L1+ stromal cells.
Primary prostate cancer [11]	Training cohort (*n* = 209) Test cohort (*n* = 611)	Semi-quantitative scoring as negative (0), weak (1), moderate (2), or strong (3)	Monoclonal rabbit PD-L1 antibody (clone EPR1161)	Moderate to high PD-L1 levels in 52.2% in the training cohort and 61.7% in the test cohort
Primary prostate cancer [12]	20	>5% membrane staining of malignant epithelial cells	5H1 clone of the mouse anti-human CD274 monoclonal PD-L1 antibody	PD-L1 positivity in 15% (3/20) of samples
Primary prostate cancer [13]	16	PD-1 positivity: negative (0), <5%; low (1+), 5–30%; high (2+), >30% of CD3+ T cells. PD-L1 staining intensity: 0 (no signal), 1+ (light signal), 2+ (high signal) in >50% of neoplastic cells.	Clone 015, Sino biological	Eight of 16 (50%) were PD-L1 positive and 19% were strongly (2+) positive
Primary prostate cancer [14]	25	“High” expression- 3 to 5 on the semiquantitative 0 to 5 score. “Low” expression- 0 to 2 on the semiquantitative 0 to 5 score	Anti-PD-L1 clone 22C3; Merck research laboratories	Low: 92% (23/25) High: 8% (2/25)

**Table 2:** Summary of resulted immune checkpoint blockade trials in prostate cancer.

NCT ID/Trial Name	Phase and Status	Disease Cohort	Number of Patients (with Prostate Cancer) Enrolled	Name of Investigational Agent	Primary Endpoint	Outcome
NCT02484404 [25]	Phase I/II Study Recruiting	mCRPC previously treated with enzalutamide and/or abiraterone	17	Durvalumab plus olaparib	Improved PFS (70% PFS vs. an estimated 50% PFS at 4 months)	rPFS of 51.5% at 12 months with a median rPFS of 16.1 months
NCT02788773 [26]	Phase II Study, active, not recruiting	mCRPC patients after prior abiraterone and/or enzalutamide, and no more than one taxane	52	Durvalumab with or without tremelimumab	ORR measured by RECIST 1.1 and iRECIST	ORR 0% (0/13) vs. 16% (6/37) and PSA response rate 0% (0/13) vs. 16% (6/37) in the durvalumab arm vs. durvalumab plus tremelimumab arm
NCT01375842 [27]	Phase I, completed	mCRPC after progression on enzalutamide and/or sipuleucel-T	15	Atezolizumab	Safety and activity	Any TRAEs 60%, one grade 3 hyponatremia, and no grade 4–5 TRAEs 12-month OS 55.6%
NCT03016312 IMbassador250 [28]	Phase III, active, not recruiting	mCRPC after the failure of an androgen synthesis inhibitor and failure of, ineligibility for, or refusal of a taxane regimen	759	Atezolizumab with enzalutamide vs. enzalutamide only	OS	Median OS 15.2 vs. 16.6 months respectively
NCT03170960 COSMIC-021 [29]	Phase 1b, recruiting	mCRPC after progression on enzalutamide and/or abiraterone	44	Cabozantinib with and without atezolizumab	ORR per RECIST 1.1	ORR per RECIST 1.1–32%
NCT03024216 [30]	Phase 1/1b, recruiting	Asymptomatic or minimally symptomatic progressive mCRPC	37	Atezolizumab and sipuleucel-T in 2 different arms (depending on the dosing schedules)	Safety and tolerability	OR by RECIST at 6 months-SD 41% (10/24) and PR 8% (2/24) Grade 3 TRAEs 12/37 (events/number of patients), Grade 4 TRAEs 2/37 (events/number of patients), no Grade 5 TRAEs or grade 3 or 4 irAEs
NCT02601014 STARVE-PC [31]	Phase 2, active not recruiting	mCRPC expressing AR-V7	15	Nivolumab plus ipilimumab	Change in PSA response (>50% PSA decline)	PSA reponse-13.3% (2/15)
NCT02985957, CheckMate 650 Trial [32]	Phase 2, recruiting	mCRPC Cohort 1 (pre-chemotherapy), cohort 2 (post-chemotherapy)	45 in cohort 1 and 45 in cohort 2	Nivolumab Plus ipilimumab	ORR at 24 weeks and Radiographic Progression-Free Survival (rPFS) at 12 months	ORR–25% and 10%, median PFS-5.5 and 3.8 months in cohort 1 and 2 respectively
NCT03338790 CheckMate 9KD, ARM B [33]	Phase II study, active, not recruiting	Chemotherapy naïve metastatic adenocarcinoma of the prostate	41	Nivolumab plus docetaxel	ORR and prostate-specific antigen (PSA) response rate (≥50% PSA reduction from baseline)	ORR–36.8% with one CR and six PRs. PSA response rate 46.3%
NCT03815942 ADVANCE [34]	Phase I/II, active, not recruiting	mCRPC patients with disease progression on enzalutamide or abiraterone	23	Viral vectored ChAd-MVA 5T4 vaccine plus nivolumab	Composite response rate measured as 50% reduction of circulating tumor DNA or 50% PSA decrease at 24-weeks	PSA (>50% PSA decrease) response at any time point 22%
NCT02489357 [35]	Pilot phase II, completed	Newly Diagnosed Oligo-metastatic Prostate Cancer	12	Pembrolizumab plus cryosurgery	Number of patients with a PSA level of <0.6 ng/mL at one year and the frequency of AEs	PSAs of <0.6 ng/mL at one year 42% (5/12) All AEs were grade ≤2
NCT02054806/KEYNOTE-28 [36]	Phase IB, active, not recruiting	PD-L1–positive heavily pretreated advanced mCRPC	23	Pembrolizumab	ORR, CR, or PR per RECIST v1.1 at any point during the study	ORR 17.4%, all 4/23 responses were PR
NCT02861573 KEYNOTE-365 COHORT A [37]	Phase 1b/2, recruiting	Docetaxel-pretreated, molecularly unselected pts with mCRPC	84	Pembrolizumab + olaparib	PSA response (>50% decline), ORR based on RECIST 1.1, number of AEs, and number of drug discontinuations due to AE’s	PSA response rate 7/82 (9%) ORR based on RECIST 1.1–2/24 (8); 2 PRs. TRAEs 83% of patients
NCT02861573 KEYNOTE-365 COHORT B [38]	Phase 1b/2, recruiting	mCRPC pts who failed or were intolerant to ≥4 wk of abiraterone or enzalutamide in the prechemotherapy setting	104	Pembrolizumab + docetaxel + prednisone	PSA response (>50% decline), ORR based on RECIST 1.1, number of AEs, and number of drug discontinuations due to AE’s	PSA response rate 29/103 (28%) ORR based on RECIST 1.1–7/39 (18%); 7 PRs TRAEs 100 (96%) of patients Grade 3–5 TRAEs 29/104 (35%) including 2 deaths from TRAEs
NCT02861573 KEYNOTE-365 COHORT C [39]	Phase 1b/2, recruiting	Chemotherapy naïve mCRPC with progression or intolerance to abiraterone	102	Pembrolizumab plus enzalutamide	PSA response (>50% decline), ORR based on RECIST 1.1, number of AEs, and number of drug discontinuations due to AE’s	PSA response rate 22% ORR based on RECIST 1.1 in patients with measurable disease 12 TRAEs 92 (90%) Grade 3–4 TRAEs 39% One treatment-related death
NCT02787005KEYNOTE-199 (cohort 1,2 &3) [40]	Phase II, active, not recruiting	mCRPC previously treated with docetaxel and targeted endocrine therapy. Cohorts 1 and 2- RECIST-measurable PD-L1–positive and PD-L1–negative disease, respectively. Cohort 3-bone-predominant disease, regardless of PD-L1 expression	258 cohort 1-133 cohort 2-66 and cohort 3-59	Pembrolizumab	ORR by RECIST 1.1	ORR was 5% in cohort 1, 3% in cohort 2
NCT02787005 KEYNOTE-199, (cohort 4&5) [41]	Phase II, active, not recruiting	Chemotherapy naive mCRPC after progression on enzalutamide, cohort 4 (RECIST-measurable disease) and cohort 5 (bone predominant disease)	126 Cohort 4-81, cohort 5-54	Pembrolizumab plus enzalutamide	ORR per RECIST v1.1 (C4)	The ORR 12% (in cohort 4), 2 CR’s and 8 PR’s
PMID: 19,147,575 [42]	Phase I, completed	CRPC with disease progression as defined by the PSA Working Group Consensus Criteria	24	Ipilimumab plus GM-CSF	AEs graded according to the National Cancer Institute Common Terminology Criteria for Adverse Events version 3.0	irAE in the higher dose cohorts-pan-hypopituitarism, mild rash, diarrhea, temporal arteritis
PMID: 17363537 [43]	Pilot trial	mCRPC	14	Ipilimumab	AEs, graded by the Common Toxicity Criteria, version 2.0	TRAEs Grade 3-asthenia, fatigue, limb pain, rash, and pruritus. No deaths or treatment discontinuation due to toxicity
NCT00113984 [44]	Phase 1, completed	mCRPC with no bone pain requiring narcotics	30	Vaccine plus GM-CSF plus ipilimumab	Safety and tolerability using NCI 3.0	The range of toxic effects exceeded those in single-agent studies especially with higher doses IrAEs were not associated with clinical responses in this study
NCT00323882 [45]	Phase I/II, completed	mCRPC	71	Ipilimumab with and without radiotherapy	AEs, prostate-specific antigen (PSA) decline, and tumor response.	8/50 patients in the 10 mg ± radiotherapy arm had PSA response (≥50% decline) and 1/28 of the tumor evaluable patients had a complete response. irAEs Grade 3–4 colitis and hepatitis and one treatment-related death
NCT01057810/(CA184-095) [46]	Phase 3, completed	Asymptomatic or minimally symptomatic patients with chemotherapy-naive mCRPC without visceral metastases	837	Ipilimumab vs. placebo	OS	Median OS 28.7 months versus 29.7 months. No improvement in OS with ipilimumab
NCT00861614/CA184-043 [47]	Phase 3, completed	mCRPC patients with progression after treatment with docetaxel	799	Ipilimumab vs. placebo following radiotherapy	OS and OS rate	Median OS 11, 2 months vs. 10, 0 months.
NCT02814669 [48]	Phase Ib, completed	mCRPC patients after progression on an androgen pathway inhibitors	45	Atezolizumab + radium-223 dichloride (r-223)	Frequency of dose-limiting toxicities and AEs. ORR per RECIST v1.1	Grade 3–4 AE’s 52.3%, 4 treatment-related deaths ORR 6.8%, no clinical benefit from combination treatment

mCRPC: Metastatic castration-resistant prostate cancer, TRAEs: Treatment-related adverse events, IrAEs: Immune-related adverse events, ORR: Overall response rate, rPFS: Radiographic progression-free survival, OS: Overall survival.

**Table 3:** Selected ClinicalTrials.gov trials involving immune checkpoint blockade in prostate cancer.

NCT Number	Phase	Number of Patients	Intervention(s)	Randomized vs. Non-Randomized	Notes
NCT03525652	Phase 1/2, recruiting	30	Therapeutic vaccine PD-1 knockout T cells	Randomized	Therapeutic vaccine—patient’s mononuclear cells are treated ex vivo with a recombinant fusion protein (PAP-GM-CSF) to induce antigen expression to activate the immune system PD-1 knockout T cells—prepared ex vivo from patient’s white cells and the maturated PD-1 knockout T cells will be infused back
NCT03658447 PRINCE	Phase Ib/II, active, not recruiting	37	177Lu-PSMA Pembrolizumab	Non-randomized	177Lu-PSMA—a compound that binds to the extracellular domain of the prostate-specific membrane antigen
NCT04631601	Phase I, not yet recruiting	105	AMG 160 Enzalutamide Abiraterone AMG 404	Non-randomized	AMG 160—BITE binds PSMA on tumor cells and CD3 on T cells AMG 404—PD-1 monoclonal antibody
NCT03689699	Phase 1b/2, recruiting	60	Nivolumab Degarelix BMS-986253	Randomized	BMS-986253—anti-IL-8 monoclonal antibody Degarelix—gonadotropin releasing hormone (GnRH) receptor antagonist
NCT03792841	Phase I, recruiting	288	AMG 160 Pembrolizumab Etanercept Immunomodulating Agent	Non-randomized	AMG 160—BITE binds PSMA on tumor cells and CD3 on T cells Etanercept—TNF-alpha inhibitor Immunomodulating Agent—prophylaxis for AMG 160-related cytokine release syndrome
NCT03910660	Phase 1b/2, recruiting	40	Talabostat Mesylate (BXCL701) plus Pembrolizumab	Non-randomized	Talabostat Mesylate (BXCL701)—a small molecule inhibitor of dipeptidyl peptidases involved in cancer progression
NCT03367819	Phase 1/2, active not recruiting	134	Isatuximab (SAR650984) Cemiplimab (REGN2810)	Randomized	Isatuximab (SAR650984)—anti-CD38 monoclonal antibody Cemiplimab (REGN2810)—anti-PD-1 monoclonal antibody
NCT02861573, (cohort G and cohort H)	Phase Ib/II, recruiting	1000 (total 10 cohorts)	MK-7684A (coformulation of pembrolizumab + vibostolimab)	Non-randomized	Vibostolimab—monoclonal antibody, that binds to the T-cell immunoreceptor with Ig and ITIM domains (TIGIT) and blocks its interaction with its ligands, CD112 and CD155, thereby activating T lymphocytes.
NCT04060342	Phase 1, recruiting	242	GB1275 nab-paclitaxel and gemcitabine pembrolizumab	Non-randomized	GB1275—CD11b modulator that reduces MDSCs and tumor-associated macrophages (TAMs), repolarizes immunosuppressive M2 tumor-associated macrophages to an M1 phenotype and increases tumor infiltration of activated CD8+ T cells
NCT04381832	Phase 1b/2, recruiting	140	Etrumadenant (AB928) Zimberelimab AB680 Enzalutamide Docetaxel	Randomized	Zimberelimab—anti-PD-1 antibody Etrumadenant(AB928)—adenosine receptor antagonist AB680- CD73 inhibitor, blocks adenosine production
NCT03493945	Phase I/II, recruiting	113	ALT-803 MVA-BN-Brachyury FPV-Brachyury Epacadostat	Randomized	M7824—bifunctional fusion protein composed of anti-PD-L1 monoclonal antibody fused with 2 extracellular domains of TGF-βRII (a TGF-β “trap”). ALT-803—a recombinant IL15 complex that delivers stimulatory signals to NK and CD8+ T cells and enhances antitumor responses Epacadostat- inhibitor of indoleamine 2,3-dioxygenase (IDO1), with immunomodulating and antineoplastic activities MVA-BN-Brachyury—priming vaccine FPV-Brachyury—boosting vaccine
NCT03629756	Phase 1, active not recruiting	44	Etrumadenant Zimberelimab	Non-randomized	Zimberelimab—anti-PD-1 antibody Etrumadenant(AB928)—adenosine receptor antagonist AB680-CD73 inhibitor, blocks adenosine production
NCT03970382	Phase 1a/1b, recruiting	148	NeoTCR-P1 adoptive cell therapy nivolumab IL-2	Non-randomized	NeoTCR-P1 adoptive cell therapy—apheresis derived CD8 and CD4 T cells that are engineered to express one autologous TCR of native sequence that targets a neoepitope presented by human leukocyte antigen (HLA) receptors exclusively on the surface of that patient’s tumor cells and not on other cells in the body.
NCT03454451	Phase 1/1b recruiting	378	CPI-006 ciforadenant pembrolizumab	Randomized	CPI-006—a humanized monoclonal antibody against CD73 cell-surface ectonucleotidase (blocks adenosine production) ciforadenant—an oral adenosine 2A receptor antagonist
NCT03829436	Phase 1/1b, recruiting	138	TPST-1120 nivolumab	Non-randomized	TPST-1120—a small molecule selective antagonist of PPARα (peroxisome proliferator-activated receptor alpha)
NCT04306900	Phase 1/1b, recruiting	152	budigalimab docetaxel mFOLFOX6 TTX-030	Randomized	Budigalimab—anti-PD-1 monoclonal antibody TTX-030-anti-CD39 monoclonal antibody that inhibits the production of adenosine
NCT04423029	Phase 1/2, recruiting	260	DF6002 Nivolumab	Non-randomized	DF6002—monovalent IL-12 immunoglobulin Fc fusion protein that establishes an inflammatory tumor microenvironment for productive anti-tumor responses
NCT03549000	Phase I/Ib, recruiting	344	NZV930 PDR001 NIR178	Non-randomized	NZV930—anti-CD73 antibody, CD73 plays a key role in the generation of extracellular adenosine PDR001-anti-PD-1 antibody NIR178-adenosine A2a receptor antagonist
NCT03849469 DUET-4	Phase 1, recruiting	242	XmAb^®^22841 Pembrolizumab	Non-randomized	XmAb^®^22841—a bispecific antibody that simultaneously targets immune checkpoint receptors CTLA-4 and LAG-3 to promote tumor-selective T-cell activation
NCT04388852	Phase Ib, recruiting	80	Ipilimumab Valemetostat	Non-randomized	Valemetostat—EZH1/2 Dual Inhibitor (stops tumor growth by blocking enzymes needed for cell growth)
NCT02643303	Phase 1/2, recruiting	102	Durvalumab Tremelimumab Poly ICLC	Non-randomized	Poly ICLC—a synthetic double-stranded RNA complex (which is a ligand for toll-like receptor-3 and MDA-5) that can activate immune cells, such as dendritic cells, and trigger natural killer cells to kill tumor cells.
NCT02655822	Phase 1/1b, recruiting	336	Ciforadenant atezolizumab	Randomized	ciforadenant—an oral adenosine 2A receptor antagonist
NCT02484404	Phase I/II, recruiting	384	Olaparib Cediranib Durvalumab (MEDI4736)	Non-randomized	Cediranib—inhibitor of vascular endothelial growth factor (VEGF) receptor tyrosine kinases
NCT04116775	Phase II, recruiting	32	Fecal microbiota transplant Pembrolizumab Enzalutamide	Non-randomized	-
